# Pancreatic Enzyme Supplementation in Acute Pancreatitis

**DOI:** 10.1155/1995/89612

**Published:** 1995

**Authors:** R. V. Patankar, R. Chand, C. D. Johnson

**Affiliations:** 1 University Surgical Unit Southampton General Hospital Southampton USA; 2 Duphar Laboratories Southampton USA; 3 CD Johnson Southampton General Hospital Southampton USA

## Abstract

This study evaluates the effect of oral pancreatic enzyme supplements on pain, analgesic requirement and the incidence of complications in patients with acute pancreatitis. This double blind, prospectively randomised placebo controlled study included 23 patients. Pain was monitored using a visual analogue scale; the analgesic requirement was assessed with a numerical score.

No significant differences were noted between the median (range) pain scores of patients who received placebo: 22 (17.1–58) and those who received enzymes: 23 (11.3–63). Hospital stay was 7 (5–10) days in patients on placebo and 8 (6–24) days in the enzyme group (*p* = 0.069). Analgesic requirements were: placebo 20 (6–60) and enzymes: 16 (0–63) (*p* = 0.56). This study has shown no beneficial effect of oral pancreatic enzyme supplements in the initial management of patients with acute pancreatitis.


*HPB Surgery*, 1995, Vol. 8, pp. 159–162
Reprints available directly from the publisher
Photocopying permitted by license only

© 1995 Harwood Academic Publishers GmbH
Printed in Malaysia

SHORT REPORT

### Pancreatic Enzyme Supplementation in Acute Pancreatitis

R. V. PATANKAR, R. CHAND* and C. D. JOHNSON

University Surgical Unit, Southampton General Hospital, *Duphar Laboratories, Southampton

This study evaluates the effect of oral pancreatic enzyme supplements on pain, analgesic requirement and the incidence of complications in patients with acute pancreatitis. This double blind, prospectively randomised placebo controlled study included 23 patients. Pain was monitored using a visual analogue scale; the analgesic requirement was assessed with a numerical score.

No significant differences were noted between the median (range) pain scores of patients who received placebo: 22 (17.1–58) and those who received enzymes: 23 (11.3–63). Hospital stay was 7 (5–10) days in patients on placebo and 8 (6–24) days in the enzyme group ($p = 0.069$). Analgesic requirements were: placebo 20 (6–60) and enzymes: 16 (0–63) ($p = 0.56$). This study has shown no beneficial effect of oral pancreatic enzyme supplements in the initial management of patients with acute pancreatitis.

KEY WORDS: Pancreatic enzymes Acute pancreatitis feedback inhibition

Feedback inhibition of pancreatic secretions by intraduodenal proteases has been demonstrated in a number of species[1–5]. Enzyme supplementation has been suggested as a means of pain relief in chronic pancreatitis but application of this approach in acute pancreatitis has not been reported previously. We postulate that intraduodenal proteases might reduce the secretory drive to the pancreas and so might reduce the severity of the pancreatitis; in animal models proglumide, a CCK receptor antagonist, has been shown to have a beneficial therapeutic effect when given after significant pancreatitis had already been initiated[6]. The aim of the present study was to evaluate the effect of pancreatic enzyme supplements on pain and the incidence of complications in patients with acute pancreatitis.

### MATERIALS AND METHODS

This study was approved by the Ethics Committee of Southampton General Hospital.

### *Patients*

Twenty three patients, (13 men) median (range) age 67 (29–86) years were entered in the study (Table 1). All patients had biochemically and radiologically proven acute pancreatitis as defined by a serum amylase level > 1000 IU/L and ultrasonographic or computerised tomography (CT) evidence of oedematous/inflamed pancreas with or without a peripancreatic collection. Patients with both mild and severe forms of acute pancreatitis were included in this study.

Only those patients who were receiving pancreatic enzyme supplements or those allergic to porcine pancreatin were excluded. None of the patients received octreotide, antisecretory agents or any other study drug in this period.

### *Study Design*

We used a double blind, prospectively randomised, placebo controlled design. All patients gave written informed consent before entering the study. Treatments were randomised by random number tables and supplied in identical containers, coded numerically.

**Address for correspondence:* CD Johnson Southampton, General Hospital, Southampton.

**Table 1** Demographic data, aetiological factors, predicted severity of disease and complications

| | *Placebo* | *Pancreatic enzymes* |
|---|---|---|
| Sex | 9 male, 5 female | 4 male, 5 female |
| Age | 68.5 (29–85) | 54 (36–86) |
| Aetiology | alcohol (6)<br>gallstones (5)<br>Unknown (2)<br>hyperparathyroidism (2)<br>steroid (1) | alcohol (6)<br>gallstones (1)<br>Unknown (3) |
| Predicted Severity | 12 mild<br>2 severe | 7 mild<br>2 severe |

Active capsules contained pancreatic enzymes as enteric coated granules packaged in gelatine capsules (Creon, Duphar Laboratories, UK). Each capsule contained 210 units free protease, 440 units zymogen bound protease, 8000 BP units lipase and 9000 BP units amylase. Placebo capsules contained microcrystalline cellulose and were identical in shape, size, appearance and taste to the active enzyme capsules. Dosage was 3 capsules 4 times a day, providing 7800 units of protease per day. Capsules were given orally; if the patient could not swallow, the capsules were opened and the granules were suspended in a small volume of water and given through a nasogastric tube. All patients had enzymes for a minimum of five days but those predicted to have a severe attack based on the modified Glasgow criteria[7] or those who developed complications received enzymes for a period of 10 days. Those patients who were discharged before completing the course of medication were given their remaining medication to take home.

Pain suffered by the patient was monitored daily using a visual analogue scale. Each patient was asked to make a mark on a 10 cm line to indicate the maximum pain he or she had suffered over the previous 24 hours. The total pain score was calculated by adding the pain scores marked by the patient on each day of their hospital stay. Intramuscular opiate analgesia was prescribed in doses related to body weight.

Other agents were prescribed according to the clinical condition of the patient. The analgesic requirements were calculated by assigning an arbitrary numerical score to the analgesics used [injected opiates 3 points; diclofenac and coproxamol (dextropropoxyphene and paracetamol) 2 points].

Length of hospital stay and the incidence of complications were also recorded. CT was performed one week after admission, and at other times as clinically indicated in all patients with complications and in those patients with predicted severe disease based on the modified Glasgow cirteria.

### *Statistical Analysis*

Power calculation suggested that this study would require 36 patients in each group assuming a 20% response in either hospital stay, pain scores or analgesic requirements with placebo and 50% with active medication in order to achieve a 5% significance with 80% power.

Data were analysed using the Mann Whitney U test.

### RESULTS

Total pain scores were not significantly different between the 2 groups, $p = 0.72$ (Table 2). No significant difference was found between the analgesic requirement scores in patients on placebo and patients on pancreatic enzymes ($p = 0.56$). Median hospital stay was not significantly different between patients on placebo and patients receiving enzymes, ($p = 0.069$). Surgery was required in 9 patients in the enzyme group and 7 in the placebo group. Five patients underwent cholecystectomy; pancreatic debridement was performed in 2 patients, one of whom also had resection of an infarcted left colon; the other patient was re-explored for drainage of a peripancreatic abscess. One patient required laparotomy to establish the diagnosis of pancreatitis and 1 patient had a cystogastrostomy followed by 2 re-explorations and packing for postoperative bleeding.

Two patients required management in the intensive care unit and one patient died. Complications arose in 5 of the 23 patients entered into the study with no difference in incidence in the 2 groups. Two patients on pancreatic enzymes developed pleural effusions with 1 of them having an associated chest infection. Three patients treated with placebo developed complications; 1 patient had an infected peripancreatic collection, 1 patient developed hypocalcemia while a third had an infarcted colon.

Significant nausea was reported by 11 of the 23 patients. This led to early breaking of the randomisation code. Nausea was reported by 7 patients who received placebo and 4 patients given enzymes. Other side effects seen in 1 patient each were diarrhoea, excessive perspiration and headache. Two patients developed symptoms and signs of alcohol withdrawal.

**Table 2** Summary of results of the analgesic scores, pain scores and the length of hospital stay

| | *Placebo Median (range)* | *Pancreatic enzyme Median (range)* |
|---|---|---|
| Analgesic score | 20 (6–60) | 16 (0–63) p = 0.56* |
| Total pain scores (mm) | 22 (17–58) | 23 (11–63) p = 0.72 * |
| Hospital stay (days) | 7 (5–10) | 8 (6–24) p = 0.69 * |

* Mann Whitney U test

Four patients failed to complete the study; one patient withdrew on the third day due to severe nausea and perspiration, 2 patients were withdrawn on the first and fourth day due to deterioration in their clinical condition and 1 patient withdrew on the third day due to symptoms of alcohol withdrawal.

Recalculation of the power estimates on the basis of the observed analgesic requirements showed that more than 150 patients would be required in both treatment groups to confirm the observed small difference in the analgesic score. Because of this and the absence of any detectable difference between the groups in complication rates, hospital stay or total pain scores that might merit evaluation in a larger series, a decision was made to terminate the study.

### DISCUSSION

In this prospectively randomised, placebo controlled double blind study, we could find no evidence of any beneficial effect of pancreatic enzyme supplements in the management of the initial stages of acute pancreatitis. No significant differences were seen in the pain scores, analgesic requirements, the length of hospital stay and the incidence of complications between the two groups (Table 2).

Trypsin dependent feedback inhibition of pancreatic secretion has been clearly demonstrated in rats[1,2,3]. These effects seem to be located in the upper intestine and mediated by cholecystokinin[3,8,9]. The presence of feedback inhibition of pancreatic enzymes by intra-duodenal trypsin in man remains controversial. In a single subject with an ampullary tumour[10], Ihse *et al.* showed a decrease in enzyme output and flow from the pancreas in response to an intra-duodenal infusion of trypsin or when the patient 's own bile and pancreatic juice was returned to the intestine. This effect was abolished when a trypsin inhibitor was added to the infusion fluid. Owyang[11] showed a dose related suppression of pancreatic output and cholecystokinin levels by an intra-duodenal infusion of trypsin.

In a model of acute haemorrhagic pancreatitis induced by feeding mice a choline deficient diet, Niederau[13] showed that proglumide (CCK antagonist) had a beneficial effect on survival and histology not only when given early in the course of acute pancreatitis but also when given after significant pancreatitis had already been initiated. This effect was reversed when intravenous CCK 8 was given in conjuction with the proglumide.

Studies in 20 patients with chronic pancreatitis[13] demonstrated suppression of pancreatic enzyme secretion by intra-duodenal infusion of proteases and a significant decrease in pain was seen in patients with chronic pancreatitis with associated exocrine insufficiency when treated with pancreatic enzymes[5,13,14,15]. On the other hand, studies by Hotz and Dlugosz[16,17] have failed to show an increase in exocrine pancreatic secretion following inhibition of intra-duodenal trypsin. Krawiscz[18] failed to demonstrate feedback inhibition of pancreatic secretion by the presence of pancreato-biliary secretions in the jejunum. More recently, Mossner[19] showed that treatment with exocrine pancreatic supplements in fact increased pancreatic enzyme secretion and was associated with elevated cholecystokinin levels. The reasons for these discrepancies include the difficulty of completely diverting pancreatic enzymes from the duodenum and of completely inactivating luminal enzymes.

It is possible that control mechanisms in healthy subjects and patients with pancreatic diseases may be different and that the feedback inhibition of trypsin may be species specific.

The failure to demonstrate an effect on pancreatic function by Hotz and Dlugosz[16,17] could have been due to the use of aprotinin as an inhibitor, which has only a limited effect on chymotrypsin and also to the use of a weak stimulus to pancreatic enzyme secretion. Furthermore, studies showing feedback inhibition in man have used pharmacological doses of trypsin[5,10,11,13]. In our study, we used approximately 7800 units of protease per day, of which the total amount of trypsin and chymotrypsin given was approximately 200–240 mg each per day, a dose commonly used in clinical practice for enzyme replacement.

We were unable to demonstrate any beneficial effect on the course of patients with acute pancreatitis with this dose of pancreatic enzymes.

Recent work in experimentally induced acute pancreatitis[20] has shown that acinar cell production of trypsinogen decreases at an early stage in the course of the disease. This may explain the failure of negative feedback with pancreatic enzymes to modify the course of acute pancreatitis in our study.

Phospholipase A2 which is implicated in the pathogenesis of acute pancreatitis is not suppressed by the protease, another possible explanation for the failure to demonstrate the beneficial effect.

### REFERENCES

1. Green, G. M., Lyman, R. L. (1972) Feedback regulation of pancreatic enzyme secretion as a mechanism for trypsin inhibitor induced hypersecretion in rats. *Proc. Soc. Exp. Biol. Med.*, **140**, 6–12.
2. Ihse, I., Lilja. P., Lundquist, I. (1972) Trypsin as a regulator of pancreatic secretion in the rat. *Scand. J. Gastroenterol.*, **13**, 873–880.

3. Schneeman, B., Lyman, R. L. (1975) Factors involved in the intestinal feedback regulation of pancreatic enzyme secretion in the rat. *Proc. Soc. Exp. Biol. Med.*, **148,** 879–903.
4. Chernick, S. S., Lepkovsky, S., Chaikoff, I. L. (1948) A dietary factor regulating the enzyme content of the pancreas: changes induced in size and proteolytic activity of the chick pancreas by the ingestion of raw soyabean meal. *Am. J. Physiol.*, **155,** 33–41.
5. Jacobson, D., Tillman, Toskes, P., Curington, C. (1982) Acute and chronic feedback inhibition of pancreatic exocrine secretion in human subjects. *Gastroenterol.*, **82,** 1091.
6. Niederau, C., Liddle, R., Ferrell, L., Grendell, J. (1986) Beneficial effects of cholecystokinin receptor blockade and inhibition of proteolytic enzyme activity in experimental acute haemorrhagic pancreatitis in mice. *J. Clin. Invest.*, **78,** 1056–1063.
7. Osborne, D. H., Imrie, C. W., Carter, D. C. (1981) Biliary surgery in the same admission for gallstone-associated acute pancreatitis. *Br. J. Surg.*, **68,** 758–761.
8. Folsch, U. R., Cantor, P., Wilms, H. M., Schafmayer, A., Becker, H. D., Cruetzfeldt, W. (1987) Role of cholecystokinin in the negative feedback control of pancreatic enzyme secretion in conscious rats. *Gastroenterology.*, **92,** 499–58.
9. Louie, D. S., May, D., Miller, P., Owyang, C. (1986) Cholecystokinin mediates feedback regulation of pancreatic enzyme secretion in rats. *Am. J. Physiol.*, **250,** 252–9.
10. Ihse, I., Lilja, P., Lundquist, I. (1977) Feedback regulation of pancreatic enzyme secretion by intestinal trypsin in man. *Digestion.*, **15,** 303–8.
11. Owyang, C., Louie, D. S., Tatum D. (1986) Feedback regulation of pancreatic enzyme secretion. Suppression of cholecystokinin release by trypsin. *J. Clin. Invest.*, **77,** 2042–7.
12. Niederau, C., Liddle, R., Ferrell, L., Grendell, J. (1986) Beneficial effects of CCK receptor blockade and inhibition of proteolytic enzyme activity in experimental acute haemorrhagic pancreatitis in mice. *J. Clin. Invest.*, **78,** 1056–1063.
13. Slaff, J., Jacobson, D., Tillman, C. R., Curington, C., Toskes, P. (1984) Protease specific suppression of pancreatic exocrine secretion. *Gastroenterology.*, **87,** 44–52.
14. Isaksson, G., Ihse, I. (1983) Pain reduction by an oral pancreatic enzyme preparation in chronic pancreatitis. *Dig. Dis. Sci.*, **28,** 97–103.
15. Halgreen, H., Pedersen, T. N., Worning, H. (1986) Symptomatic effect of pancreatic enzyme therapy in patients with chronic pancreatitis. *Scand. J. Gastroenterol.*, **21,** 104–108.
16. Hotz, J., Ho, S. B., Go, V. L. W., DiMango, E. P. (1983) Short-term inhibition of duodenal tryptic activity does not affect human pancreatic, biliary or gastric function. *J. Lab. Clin. Med.*, **101,** 488–95.
17. Dlugosz, J., Folsch, U. R., Creutzfeldt, W. (1983) Inhibition of intraduodenal trypsin does not stimulate exocrine pancreatic secretion in man. *Digestion.*, **26,** 197–204.
18. Krawisz, B. R9., Miller, L. J., DiMango, E. P., Go, V. L. W. (1980) In the absence of nutrients, pancreatic-biliary secretions in the jejunum do not exert feedback control of human pancreatic or gastric function. *J. Lab. Clin. Med.*, **95,** 13–8.
19. Mossener, J., Wresky, H., Zeeh, J., Regner, U., Fischbach, W. (1989) Influence of treatment with pancreatic extracts on pancreatic enzyme secretion. *Gut*, **30,** 1143–1149.
20. Iovanna, Keim, V., Michel, R., Dagorn, J. (1991) Pancreatic gene expression is altered during acute experimental pancreatitis in the rat. *Am. J. Physiol.*, **261,** G485–G489.

